# Combined antegrade femur lengthening and distal deformity correction: a case series

**DOI:** 10.1186/s13018-020-02168-6

**Published:** 2021-01-15

**Authors:** Achraf Jardaly, Shawn R. Gilbert

**Affiliations:** 1 Gilbert and Rose-Marie Chagoury School of Medicine, Lebanese American University, Byblos, Lebanon; 2 Department of Orthopedic Surgery, University of Alabama at Birmingham, Birmingham, AL USA; 3 Department of Pediatric Orthopaedics, ACC Suite 316, Children’s Hospital of Alabama, 1600 7th Avenue South, Birmingham, AL 35233 USA

**Keywords:** Leg length discrepancy, Combined femoral deformities, Limb lengthening, PRECICE nail, Magnetic intramedullary nailing, Two-level osteotomy

## Abstract

**Background:**

Leg length discrepancy is often associated with distal femur angular deformities such as valgus or flexion. This study aims to report a new technique for simultaneous limb lengthening and acute distal femoral angular correction.

**Methods:**

A retrospective chart review of patients undergoing a single procedure was conducted. Patients included had a single operation where they underwent distal femur osteotomy stabilized with a plate followed by antegrade nailing with a magnetically controlled intramedullary lengthening nail (PRECICE, Ellipse Technologies, Inc., Irvine, CA, USA) using a trochanteric entry.

**Results:**

Seven femurs from 7 patients were included. The average age at operation was 13.6 years, and the leg length difference was 51 mm (range 30–105 mm). Associated deformities were valgus (4), knee flexion contracture (2), and both valgus and flexion contracture (1). Lengthening achieved was 43 mm (*P* = 0.0036), with a consolidation index of 27 days/cm and reliability of 0.87 (6/7). The 5 patients with angulation had an improvement of valgus from 12 to 4° (*P* = 0.006) and of the mechanical axis deviation from 34 to 3 mm (*P* = 0.0001). The range of motion also improved in the 3 patients with contractures. Preoperative gait disturbance, hip and knee pain, and functional scoliosis resolved after the limb deformities were corrected.

**Conclusion:**

Combining a magnetic internal lengthening nail with a second distal osteotomy stabilized with a plate can successfully correct limb length and distal femur deformity acutely without altering the expected result of each procedure.

## Background

Abnormalities in the femur and tibia are the leading cause of leg length difference (LLD). These abnormalities commonly stem from congenital, infectious, or traumatic conditions [1]. While a variety of conservative and operative options exists, limb lengthening procedures are increasingly preferred due to technical advances and patient preference [2]. These procedures have drastically changed with the advent of intramedullary lengthening rods. Nonetheless, osteogenesis by distraction as described by Ilizarov is still how lengthening is achieved [3]. Internal lengthening nails (ILN) have the advantage of eliminating the need for external fixators and their associated complications [4–6]. Among the lengthening devices, magnetic intramedullary nails offer the most accurate distraction and precise lengthening [1, 2, 4, 6–11].

Magnetic ILN have advanced limb reconstruction, greatly achieving higher patient satisfaction, more reliable outcomes, and fewer complications than other lengthening methods [2, 4, 12, 13]. However, patients with combined deformities still remain a challenging group, and they require multiple operations. Short limbs often also have angular deformities [14]. Deformities in the distal femur can exacerbate patients’ LLD and could need to be surgically addressed. As such, additional procedures might be needed over what is already a technically challenging operation with a long healing process. Simultaneous lengthening and significant deformity correction are considered a contraindication to magnetic ILN by some authors [4].

Few reports exist on concomitant procedures to address combined deformities. They demonstrate adequate correction of angular deformities and LLD using one operation, but an external fixator is typically used [14–16]. Most commonly, retrograde nailing with blocking screws is used for distal femur deformities [14, 17]. To avoid complications associated with this technique, here, we report our experience with a less common approach in the correction of different distal femur angular deformities accompanying LLD: antegrade nailing and the use of a distal femoral osteotomy (DFO) stabilized with a plate [18].

This manuscript aims to advance clinical outcomes and can be considered to fit in the framework of translational orthopedics. This is in concordance with an increasing number of publications attempting to advance orthopedic practice [19].

## Methods

This is a retrospective review conducted at a single institution following Institutional Review Board approval (University of Alabama at Birmingham IRB-300004224 on March 2020). Patients undergoing concurrent femur lengthening and distal femoral osteotomy were included. Charts were reviewed for patient demographics, etiologies of the deformities, indications for surgery, operative details, and clinical findings and complications documented in clinical visits. The Paley classification of difficulties during lengthening procedures (problems, obstacles, and true complications) was used [20]. Problems are difficulties that resolve without operative intervention, obstacles require reoperation, and true complications are intraoperative injuries as well as problems that persist after the treatment ends. The reliability of lengthening was determined as defined by Schiedel et al: as the ratio of the number of successful treatments achieving bony consolidation to the number of total implants [7]. Pre- and postoperative radiographs were used to determine the leg length difference, the valgus deformity, the mechanical axis deviation (MAD), the lateral distal femoral angle (LDFA), and the medial proximal tibial angle (MPTA). Student *t* tests were used to compare measurements before and after the surgery, with the threshold of statistical significance set at a two-tailed *P* value of 0.05 [21].

### Surgical procedure

The senior author performed all surgeries at a single institution. The operation was started by performing the DFO. A lateral approach was used, and with the assistance of a guide wire, a wedge osteotomy was performed. The osteotomy was then stabilized with a plate.

**Fig. 1:**
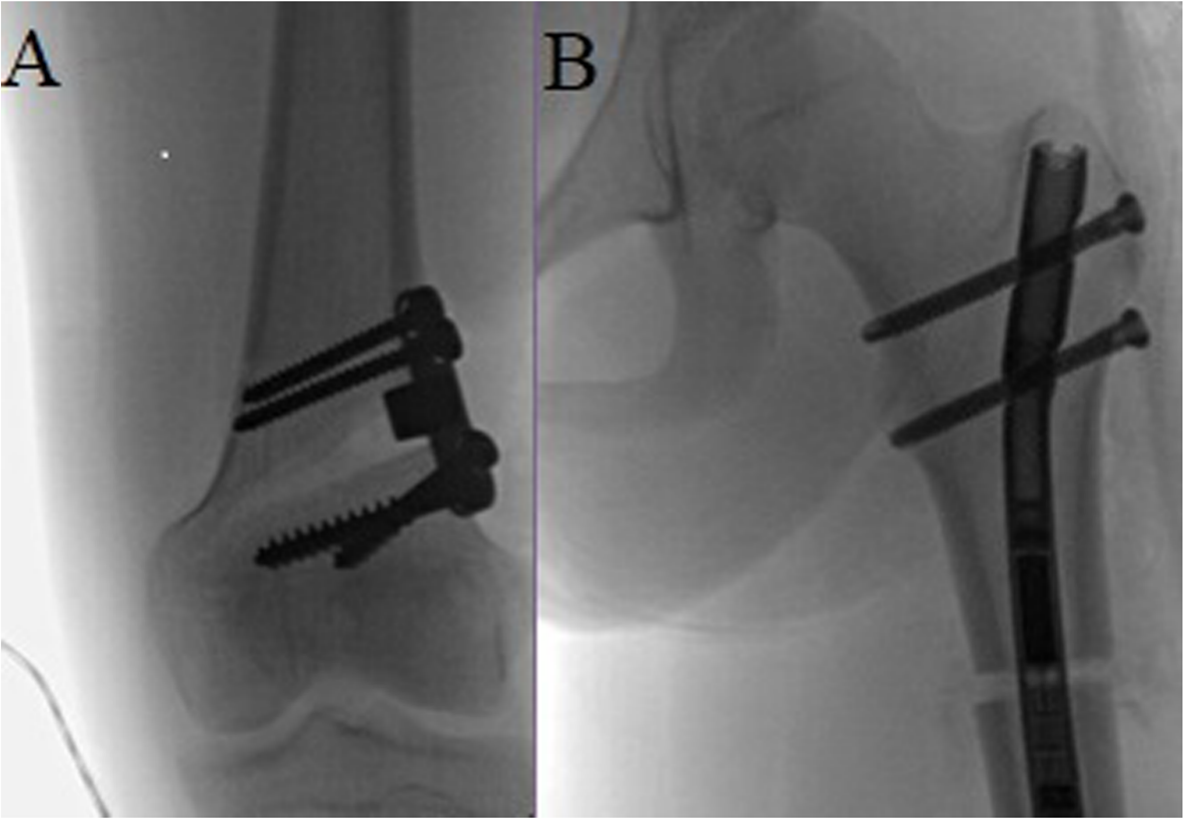
Intraoperative fluoroscopy demonstrates **a** stable distal femur osteotomy and **b** functioning lengthening nail with adequate distraction

**Fig. 2:**
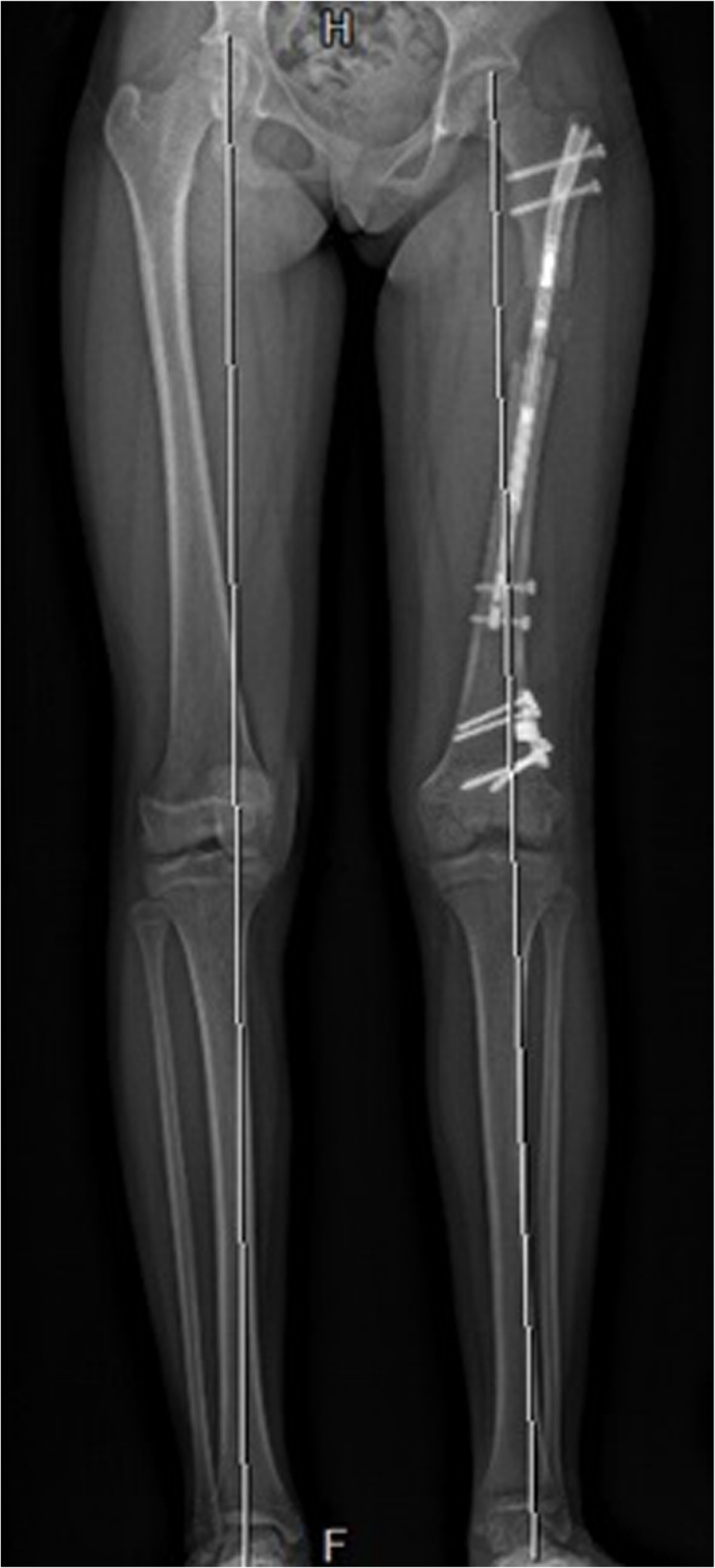
X-ray at a follow-up visit demonstrates interval lengthening and good alignment of the weight-bearing axis

An antergrade, magnetically controlled intramedullary lengthening nail (PRECICE, Ellipse Technologies, Inc., Irvine, CA, USA) was inserted in the established technique using a trochanteric entry point [22]. Fluoroscopy confirmed adequate placement of the hardware and functioning of the nail intraoperatively (Fig. 1). The nail was tested intraoperatively for proper lengthening using an external remote control magnet. Patients were discharged within 1 to 3 days of the procedure. Lengthening was started on postop days 4 to 8, at a rate of 1 mm/day over 3 sessions. Follow-up visits were performed at 1- to 2-week intervals to monitor the range of motion, and lengthening and alignment were verified by radiographs (Fig. 2).

## Results

Since the introduction of the PRECICE nail in December 2011, 32 patients underwent a femur lengthening procedure at our institution, 20 of which utilized the magnetic ILN. Seven patients (7 femurs) underwent concurrent lengthening with a magnetic ILN and DFO. The average age at operation was 13.6 years (Table 1). DFO was performed to correct excessive valgus (4 patients), flexion deformity (2), or both deformities (1).

**Table 1:** Patient demographics and operative details

Variable	Value
Age*, years	13.6 (10.75–16.2)
Sex, number	
Male	2
Female	5
Weight*, kg	58.1 (43.4–73.2)
Laterality, number	
Right	4
Left	3
Previous lengthening operations, number	
0	2
1	3
2 or more	2
Cause, number	
Congenital femoral deficiency	2
Meningococcemia	2
Russell-Silver syndrome	1
Ollier’s syndrome	1
Idiopathic	1
Associated deformity, number	
Valgus	4
Knee flexion contracture	2
Both valgus and knee flexion contracture	1
Blood loss*, mL	246 (50–500)
Surgery time*, h:min	3:30 (2:16–5:02)
Follow-up duration*, months	22 (8–49)

*Each value is expressed as the mean, with the range in parenthesis

All patients had LLD ≥ 30 mm and were symptomatic (Table 2). By the final follow-up visit, these symptoms had resolved except for one patient complaining of mild knee pain. Six patients healed without complications, while 1 patient experienced osteomyelitis and fixation failure 15 days postop. Of note, she had previous lengthening and prior intramedullary nail placement with surrounding sclerosis along a path other than the desired one for the lengthening. This made creating and reaming a new path for the magnetic ILN very challenging. She underwent debridement and nail replacement. Therefore, we had 1 obstacle, and the reliability was 0.86 (6/7).

**Table 2:** Symptoms of patients before and after surgery

	Pre-surgery, number (%)	Post-surgery, number (%)
Gait abnormality	4 (57%)	0
Knee pain	3 (43%)	1 (14%)
Back pain	2 (28%)	0
Functional scoliosis	2 (28%)	0

The average lengthening achieved was 43 mm (Table 3). No premature or delayed consolidation was observed, and the consolidation index was 27 days/cm on average. For the 5 patients with valgus, the average angulation was 12°, which was mostly due to femoral deformity (Table 3). Valgus and mechanical axis deviation were corrected by 8° (*P* = 0.006) and 31 mm (*P* = 0.0001), respectively.

**Table 3:** Length and angular deformities

	Pre-surgery		Post-surgery		*P* value
	Mean ± SD	Range	Mean ± SD	Range	
LLD (*n* = 7), mm	51 ± 26	30–105	8 ± 8	1–23	0.0036
Angulation (*n* = 5)					
Valgus, degrees	12 ± 4	7–16	4 ± 2	2–7	0.006
MAD, mm	34 ± 8	27–45	3 ± 5	0–11	0.0001
LDFA, degrees	82 ± 8	72–94	87 ± 4	80–90	0.28
MPTA, degrees	92 ± 7	82–99	90 ± 2	87–91	0.60

*LDFA* lateral distal femoral angle, *LLD* limb length discrepancy, *MAD* mechanical axis deviation, *MPTA* medial proximal tibial angle, *SD* standard deviation

Three patients initially had knee flexion deformity. They improved clinically and reached their target range of motion (Table 4). The four remaining patients regained their full knee range of motion in less than 4 months following the index procedure.

**Table 4:** Lack of extension for patients with flexion deformity

	Pre-surgery (°)	Post-surgery (°)	Time to maximal extension (months)
Patient 1	12	0	1
Patient 2	12	0	12
Patient 3	45	15*	11

*Intentionally left slight flexion deformity due to limited arc of motion secondary to condylar dysplasia

## Discussion

**Fig. 3:**
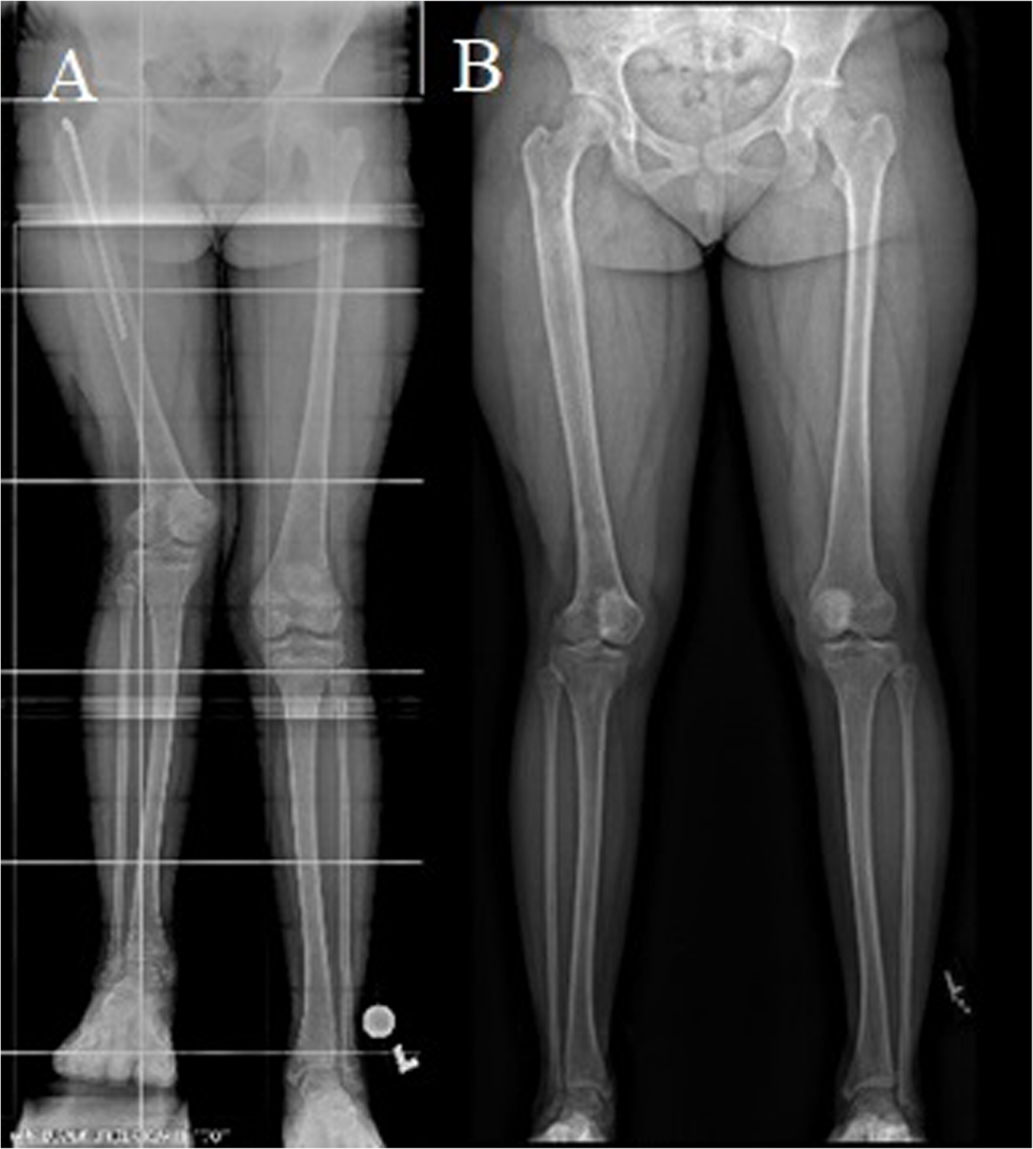
Patient with an acquired leg length discrepancy and 2 prior lengthening operations for his right femur. **a** Right femur shortening and valgus deformity preoperatively. Patient is standing on a 6-cm block. **b** Significant improvement of both deformities at final follow-up

In this paper, we report our experience in combining antegrade nailing and a plate-stabilized DFO to target LLD and valgus and/or flexion contractures in seven patients. Our patients achieved their planned lengthening and had a clinically significant correction of their other deformities (Fig. 3). Preoperative gait disturbance, hip and knee pain, and functional scoliosis resolved in all but one patient after the limb deformities were corrected. The one patient with knee pain postoperatively reported great improvement in the pain compared to before the surgery. No cases of avascular necrosis occurred, confirming previous findings regarding the safety of femoral lengthening via a trochanteric entry in children [23].

The consolidation index of our patients was 27 days/cm, comparable with the values between 31.6 and 33.6 days/cm reported in the literature. This indicates that bone healing was not affected and that consolidation can be expected to occur as normally anticipated without delay. Similarly, the reliability of our lengthening was 0.86. Magnetic ILN used in patients with LLD typically have a reliability between 0.78 and 0.85 due to the challenges posed by this patient population [7, 8, 24–26]. Furthermore, deformity correction in our patients was successful in terms of desired length and angulation correction. After the surgery and at a lengthening rate of 1 mm/day, the mean LLD was only 8 mm, with an average correction of 43 mm. This correction is in line with the usual lengthening achieved using the PRECICE nail, which has commonly reported lengthening averages between 35 and 44 mm [7–9, 18]. Achieved MAD and LDFA correction in patients with valgus were 18.3 mm and 5.8°, respectively, in a prior study using an external fixator in conjunction with an ILN to achieve simultaneous femoral lengthening and deformity correction [14]. Our results were similar, with MAD correction of 31 mm and LDFA correction of 5°. Our favorable outcomes suggest that a DFO does not compromise the lengthening and healing processes as indicated by the reliability and consolidation index, respectively. Though this results in a longer operation, the advantages of a combined procedure offset the increased surgery time. Such advantages include avoiding a 2nd procedure with general anesthesia and subsequent recovery time and postoperative pain, reducing cost, and decreasing hospitalization [15]. As a result, the simultaneous correction of multiple femoral deformities is expected to be used more frequently. Several techniques can achieve this. These usually involve retrograde nailing and the use of distal blocking screws. Steiger et al. demonstrate the success of using a two-level osteotomy with retrograde nailing in 2 femurs [17]. Our technique with antegrade nailing provides a viable alternative that avoids violating the knee while still allowing accurate nail placement and precise distraction. The advantage of antegrade nailing over a retrograde approach is its ability to be used in skeletally immature patients and in children with a narrow canal [22]. This advantage is highlighted in patients with congenital deformities who might benefit from early interventions. In addition, retrograde nailing can itself increase flexion deformity and varus and valgus angulation in patients, thereby exacerbating the additional deformities present [18]. In antegrade nailing, less restriction of the knee movement can be expected [24]. Furthermore, blocking screws with nailing are commonly employed with nailing to address the distal femur deformity in conjunction with limb shortening. Iobst and colleagues demonstrated the success of this approach which resulted in the correction of both the lateral distal femoral angle and the LLD in their cohort [14]. However, the use of blocking nails may be more technically challenging, whereas using a plate for stability and a trochanteric entry for nailing employs procedures more frequently performed by pediatric orthopedic deformity surgeons. The use of a nail and plate does theoretically create a stress riser between the implants, but we did not encounter any periprosthetic fractures. Future biomechanical studies could provide insight into this potential risk.

In light of the advantages of using a single procedure with an antegrade ILN and a DFO stabilized with a plate, we use this procedure to treat shortening and distal femur deformities. This technique was described by Fragomen and Fragomen who report their experience in treating three patients [18]. Satisfactory limb lengthening and distal deformity correction were achieved. The patients had knee flexion contractures in addition to short limbs. This was due to the traumatic closure of the distal femoral physis. We had similar outcomes in our cohort of congenital and acquired deformities, suggesting that this technique holds reliability in correcting limb length and distal femur deformities of different etiologies. Regarding the knee range of motion in children with flexion deformity, extension gained was between 12 and 30° in our patients, similar to the 15° reported by the aforementioned authors [18].

Adequate distraction and subsequent lengthening were observed in 5 patients. Despite being compliant, the remaining 2 patients had a slower lengthening rate than anticipated. This was addressed by advising them to assume a lateral decubitus position during lengthening to minimize soft tissue interference with magnet engagement, which resolved their slow progress. Nonfunctional distraction is a possible complication of using a magnetic lengthening nail, with rates of 4% being reported [8]. In the setting of slightly slow lengthening despite adequate instrumentation and use, similar conservative approaches could be tried before resorting to revision surgery.

We acknowledge the limitations of the present study. An uncommon procedure was studied, so the number of patients was small. This is common in similar articles discussing novel techniques for deformity correction, which are typically small case series [17, 18]. Retrospective review also has the risk of suffering from inaccuracy and inconsistency. However, since the procedures were performed by the senior author at a single institution, variability in the surgical procedure, patient interviews, and documentation could be minimized. No patient-reported outcomes were included. Though we attempted contacting patients to acquire functional scores, only scores from two patients were obtained. This was also limited by the absence of preoperative functional scores, so we did not include these outcomes. Nonetheless, this study still highlights a readily implementable technique for acute deformity correction as well as lengthening using a magnetic ILN, and it also shows that successful correction of the individual deformities can be achieved reliably and with minimal complications.

## Conclusion

In summary, a single procedure achieving acute distal femoral angular correction and limb lengthening can be performed using a magnetic ILN and a distal femur plate, without the need for external fixation. Antegrade femur lengthening can be employed, and combining lengthening with a DFO does not appear to alter the expected result of each operation.

Future studies can help generalize our conclusion to encompass more patients and conditions, and they can have longer follow-up times into adulthood to investigate long-term results and possible complications. Moreover, patient-reported functional outcomes are pivotal to assess how patients and caregivers perceive this operation as compared to more traditional, multi-staged procedures.

## Data Availability

Not applicable

## Abbreviations

DFO: Distal femoral osteotomy
ILN: Internal lengthening nail
KOOS: Knee Injury and Osteoarthritis Outcome Score
LDFA: Lateral distal femoral angle
LEFS: Lower extremity functional scale
LLD: Leg length difference
MAD: Mechanical axis deviation
MPTA: Medial proximal tibial angle
QOL: Quality of life
